# Supplementary material for: Transgene codon usage drives viral fitness and therapeutic efficacy in oncolytic adenoviruses
Source: NAR Cancer. 2021 Apr 26;3(2):zcab015. doi: 10.1093/narcan/zcab015 (PMC8210037; doi:10.1093/narcan/zcab015)
Supplement: zcab015_Supplemental_File [file zcab015_supplemental_file.docx]

**Transgene codon usage drives viral fitness and therapeutic efficacy in oncolytic adenoviruses**

Estela Núñez-Manchón, Martí Farrera-Sal, Marc Otero-Mateo,Giancarlo Castellano, Rafael Moreno, David Medel, Ramon Alemany, Eneko Villanueva, Cristina Fillat.

**Supplementary Information**

**Supplementary Figure S1: Transgenes expressed in OAds currently in clinical trials use suboptimal codons.**

**(A)** Codon Adaptation (CAI) analysis of clinical trial OAds transgenes in comparison with 700 randomly selected human proteins (grey) with a men CAI value of 0.80. Red and black vertical lines correspond to the CAI value of clinical trials OAds transgenes cassettes (with available sequence) expressed in early and in late viral phase, respectively. **(B)** CAI analysis of the enhanced green fluorescent protein (*EGFP)* and the low codon optimised GFP (*LGFP*).

**Supplementary Figure S2: Transgene codon usage does not impact viral fitness when expressed at early phase of infection.**

**(A)** Schematic representation of the whole adenoviral genome armed with EGFP or LGFP transgenes in late (under the MLP control downstream the L5-Fiber transcription unit) and in early (under the control of CMV constitutive promoter inserted between E4 and the right ITR). **(B)** Proliferation assay in A549 cells infected with 5 IFU of Adwt-EGFP_late, Adwt-LGFP_late, Adwt-EGFP_early, Adwt-LGFP_early or Adwt, analyzed by Incucyte live cell motorization during a period of 100h. Data is represented as the mean ±SEM of four experimental replicates. **(C)**Fluorescence assay in A549 cells infected with 5 IFU of Adwt-EGFP_late, Adwt-LGFP_late, Adwt-EGFP_early or Adwt-LGFP_early, analyzed by Incucyte live cell motorization during a period of 100h. Data is represented as the mean ±SEM of five independent experimental replicates.

**Supplementary Figure S3: Hybrid transgenes obtained from EGFP and LGFP sequences express GFP proportionally to their codon optimization in terms of GC3 content.**

**(A)** Schematic model of chimeric GFP transgenes *CH1*, *CH2* and *CH3* design. **(B)** Schematic model of all GFP transgenes: the enhanced green fluorescent protein EGFP, the low codon optimised green fluorescent protein LGFP and the chimeric GFPs CH1, CH2 and CH3. **(C)** Global Codon Adaptation Index (CAI) analysis according to the human codon usage of GFP transgenes in comparison to early regulatory adenoviral genes (in blue) and to virus structural and replication involved genes (in yellow). **(D)** CAI along the sequence; line pattern represents smoothened CAI values along the sequence and the numeric value corresponds to the global CAI value for each sequence. **(E)** Codon usage optimization analysis in terms of GC3% of GFP transgenes in comparison to early regulatory adenoviral genes (in blue) and to virus structural and replication involved genes ( in yellow). **(F)** Transgenes codon usage evaluation by Principal Component Analysis (PCA): Loadings in the left panel showing codons coloured according to the 3^rd^ nucleotide composition; scores in the right panel showing the distribution of all viral genes as well as *EGFP,* *LGFP, CH1, CH2* and *CH3* transgenes in the first two principal components (PC1 and PC2). Early adenovirus regulatory genes are represented in light grey, with an increased usage of AT3 codons (orange and red spheres). Late structural and replication genes are represented in dark grey, with an increased usage of GC3 codons (grey and blue spheres). **(G)** Fluorescence analysis by flow cytometry of 293T cells 48h post-transfection with miRVec-EGFP, miRVec-CH1, miRVec-CH2, miRVec-CH3 and miRVec-LGFP expression plasmids. Data is represented as box plot of three independent experiments.

**Supplementary Figure S4: Bee hyaluronidase (BWT) activity is higher than human hyaluronidase PH20.**

**(A)** Equivolumes of supernatants from 5-days HEK293 cells transfected with GT4082-BWT or GT4082-hPH20 plasmids were assessed by anti-HisTag Western Blot. A commercial purified recombinant His-tagged hPH20 (Acro Biosystems, PH0-H5225) was used as a positive control to perform a standard curve ranging from 20 to 80 ng in order to quantify the samples. **(B)** Hyaluronidase activity in the supernatants of the 5 days HEK293 transfected cells was analysed by turbidimetric assay and normalised according to the amount of protein detected in Western Blot. Representative results from one of three experiments are shown. Bars represent the mean±SD of triplicates. **p-value<0.01 by unpaired two-tailed T-test.


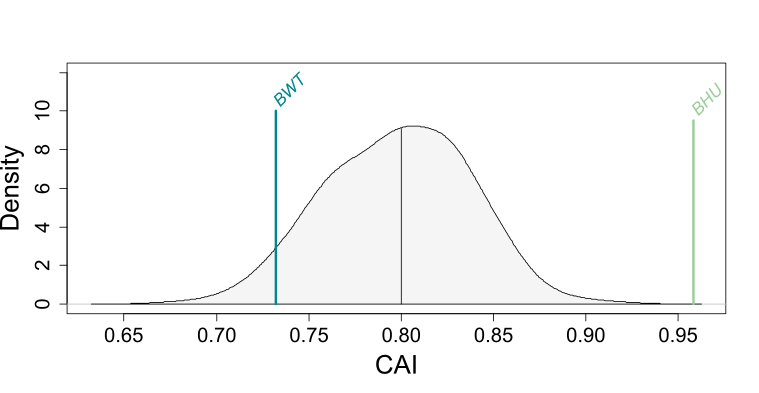


**Supplementary Figure S5:**

CAI analysis of the wild-type hyaluronidase (*BWT*) and the codon humanised bee hyaluronidase (*BHU*), in relation to human genes CAI.

**Supplementary Figure S6: Codon humanised bee hyaluronidase transgene (*BHU*) impairs viral oncolytic activity.**

**(A)** qPCR relative quantitation of the extracellular viral particles released to the supernantants of MIA PaCa-2 cells infected with 5 IFU of VCN-01, ICO15K-BHU or ICO15K-BWT at 72 hpi. The dashed line represents VCN-01 values. Data is represented as the mean ±SEM; each dot corresponds to an independent experimental replicate. *p<0.05 (two tailed Mann- Whitney test). **(B-C)** *In vitro* oncolytic activity assay in MIA PaCa-2 **(b)**and NP18 *(c)* cells. Cells were infected with a dose range of ICO15K-BHU and ICO15K-BWT and their viability was measured 7 days PI by MTT assay. Viability curves are represented in the upper panel. Data is represented as mean ±SEM for at least four independent experiments. The lower panel shows IC_50_ values calculated from viability curves. Data is represented as box plot of four independent experiments. *p<0.05 (two tailed Mann- Whitney test).

**Supplementary Figure S7: Codon humanised FBiTE transgene (*FBiTEHU*) impairs viral oncolytic activity.**

**(A)** Principal Component Analysis of the relative codon usage per amino acid of FBiTE and FBiTEHU transgenes: the left panel shows the loadings, codons characterised according to the 3^rd^ nucleotide composition, the right panel shows the FBiTE transgenes (red) in relation to adenoviral genes. **(B)** Schematic representation of ICO15K-FBiTEHU and ICO15K-FBITE virus, armed with codon humanised FBiTE (*FBiTEHU*) and codon wild type FBiTE respectively. **(C)** qPCR relative quantitation of the extracellular viral particles released to the supernantants of A549 cells infected with 30 IFU and 0.5 IFU at 36h and 72h respectively. Data is represented as the mean ±SEM; of at least 6 independent replicates. **p<0.01 (two tailed Mann- Whitney test). **(D)** Up: Representative bright-field images of A549 cell 72h after infection with ICO15K-FBiTEHU (middle) or ICO15K-FBiTE (right) at a 5 IFU/cell (left, A549 un-infected as control). Down: quantification of FBiTE expressed from ICO15K-FBiTEHU or ICO15K-FBiTE infected A549 cells. CD3+ Jurkat cells were incubated with supernatant from non-infected (mock) or infected (ICO15K-FBiTEHU or ICO15K-FBiTE) A549 cells (72h after infection), and FBiTE binding was determined by flow cytometry using an anti-FLAG-FITC antibody. **(E)** *In vitro* oncolytic activity assay in A549 cells. Cells were infected with a dose range of ICO15K-FBiTEHU or ICO15K-FBiTE and their viability was measured 5 days PI by MTT assay. Results are represented as mean ±SEM of at least four independent experiments. *p<0.05 (two tailed Mann- Whitney test).

**Supplementary Figure S8: Modulating codon optimization of bee hyaluronidase transgene leads to optimal therapeutic index response in MIA PaCa-2 *in vivo* model.**

**(A)** *In vivo*  tumour growth assay in mice bearing subcutaneous MIA PaCa-2 tumours. Animals were intravenously treated with saline solution or 4·10^10^ vp/animal of either VCN-01, ICO15K-BHU, ICO15K-BWT or ICO15K-BAd (n≥6 animals/group). Follow-up of tumour volumes is represented as mean of percentage of growth ±SEM. **(B)** Tumour weight at end-point. **(C)** Tumour volume at end-point. **(D)** HA acid staining quantitation of deparaffinised tumour sections with HABP. Scale bar 100 µm. Left panels: representative images of HA staining. Right panels: HA acid quantification representing stained area in at least 5 fields from the tumour sections of at least 3 different mice. **(E)** Tumour cellularity measured by Masson staining. Scale bar 100 µm. Left panels: representative images of HA staining. Right panels: HA acid quantification.

*p<0.05, **p<0.01, ***p<0.001, # p<0.05, ## p<0.01, $ p<0.05, $$ p<0.01 * represents statistical differences in relation to saline group; # represents statistical differences in relation to saline VCN-01 group, $ represents statistical differences in relation to ICO15K-BHU group.

**Supplementary Figure S9: Uncropped images of western blot figures shown in the main paper.**

**Supplementary Table S1. Primer sequences**

| **Primer set** | **Primer name** | **Primer Sequence** |
| --- | --- | --- |
| **1** | Fw_BamHI_EGFP | TATGCGGATCCATGGTGAGCAAGGGCGAGGAGC |
|  | Rv_EcoRI_EGFP | TATGCGAATTCTTACTTGTACAGCTCGTCCATGC |
| **2** | Fw_BamHI_pMONO-neo-GFP | TATGCGGATCCATGAGCAAGGGAGAAGAACTC |
|  | Rv_EcoRI_pMONO-neo-GFP | TATGCGAATTCTTACTTGTACAGCTCATCCATTCC |
| **3** | sPH20_Fwd | CCACCGGTGCCACCATGGG |
|  | sPH20_Rev | AACGCGGCCGCTTTATTAGTGGTGGTGGTGGTGGTGGGGGCCCTGGAACAGCACCTCCAGAGATAGTGTGGAGGGTGAAGC |
| **4** | BHyal_Fwd | ATCCACCGGTCCACCATGTCTCGGCCTCTCGTGAT |
|  | BHyal_Rv | CGCGGCGGCCGCTTTAGTGGTGGTGGTGGTGGTGGGGGCCCTGGAACAGCACCTCCAGCACTTGGTCCACGCTCACGTC |
| **5** | Fw_Ad_late_pMONO-neo-GFP | cgtgtttatttttcaattggtactaagcggtgatgtttctgatcagccaccATGAGCAAGGGAGAAGAACTC |
|  | Rv_Ad_late_pMONO-neo-GFP | gaatgaaaaatgacttgaaattttctgcaattgaaaaataaagtttattaTTACTTGTAC AGCTCATCCATTCC |
| **6** | Recombination BHU FW | atcgtttgtgttatgtttcaacgtgtttatttttcaattggtactaagcggtgatgtttctgatcagccaccATGTCTCGGCCTCTCGTGA |
|  | Recombination BHU RV | gctatactactgaatgaaaaatgacttgaaattttctgcaattgaaaaataaagtttattaCACTTGGTCCACGCTCA |
| **7** | Fiber-GFP Fw | caattggtactaagcggtgatgtttctgatcagccaccATGGTGAGCAAGGGCGAGG |
|  | Fiber-GFP Rv | gacttgaaattttctgcaattgaaaaataaagtttattaCTTGTACAGCTCGTCCATGC |
| **8** | E4 miARN cua Fw | GAAAACTACAATTCCCAACACATACAAGTTACTCCGCCCTAACTTTTATTTTATCGAATCT GC |
|  | E4 miARN cua Rv | CGTGGCGCGGGGCGTGGGAACGGGGCGGGTGACGTAGGTTACATTGATTATTGACTAG |
| **9** | qPCR-Ad-genome-Fw | GCCGCAGTGGTCTTACATGCACATC |
|  | qPCR-Ad-genome-Rv | CAGCACGCCGCGGATGTCAAAG |
| **10** | qPCR-hexon-Fw | GTCTACTTCGTCTTCGTTGTC |
|  | qPCR-hexon-Rv | TGGCTTCCACGTACTTTG |
| **11** | qPCR-fiber-Fw | CTCCAACTGTGCCTTTTC |
|  | qPCR-fiber-Rv | GGCTCACAGTGGTTACATT |
| **12** | qPCR-ACTB-Hs-Fw | CTGGAACGGTGAAGGTGACA |
|  | qPCR-ACTB-Hs-Rv | GGGAGAGGACTGGGCCATT |
| **13** | Recombination FBiTEHU-Fw | caattggtactaagcggtgatgtttctgatcagccaccATGGGCTGGTCCTGCATCATC |
|  | Recombination FBiTEHU-Rv | gacttgaaattttctgcaattgaaaaataaagtttattaCTTGTCATCGTCGTCCTTGTAG |

**Supplementary Data S1. GFP sequence alignments**

**Gene sequence alignment**

EGFP ATGGTGAGCAAGGGCGAGGAGCTGTTCACCGGGGTGGTGCCCATCCTGGTCGAGCTGGAC 60

LGFP ---ATGAGCAAGGGAGAAGAACTCTTTACTGGTGTTGTCCCAATTCTGGTTGAGCTGGAT 57

********** ** ** ** ** ** ** ** ** ** ** ***** ********

EGFP GGCGACGTAAACGGCCACAAGTTCAGCGTGTCCGGCGAGGGCGAGGGCGATGCCACCTAC 120

LGFP GGTGATGTGAATGGCCACAAATTCTCTGTGTCTGGTGAAGGTGAAGGAGATGCAACTTAT 117

** ** ** ** ******** *** ***** ** ** ** ** ** ***** ** **

EGFP GGCAAGCTGACCCTGAAGTTCATCTGCACCACCGGCAAGCTGCCCGTGCCCTGGCCCACC 180

LGFP GGAAAGCTGACTCTGAAGTTCATTTGTACAACAGGAAAGCTGCCAGTGCCTTGGCCAACT 177

** ******** *********** ** ** ** ** ******** ***** ***** **

EGFP CTCGTGACCACCCTGACCTACGGCGTGCAGTGCTTCAGCCGCTACCCCGACCACATGAAG 240

LGFP CTGGTGACCACCCTGACTTATGGTGTTCAATGTTTCAGCAGGTACCCTGACCACATGAAG 237

** ************** ** ** ** ** ** ****** * ***** ************

EGFP CAGCACGACTTCTTCAAGTCCGCCATGCCCGAAGGCTACGTCCAGGAGCGCACCATCTTC 300

LGFP CAGCATGACTTCTTTAAATCTGCAATGCCAGAAGGTTATGTTCAGGAGAGGACAATCTTC 297

***** ******** ** ** ** ***** ***** ** ** ****** * ** ******

EGFP TTCAAGGACGACGGCAACTACAAGACCCGCGCCGAGGTGAAGTTCGAGGGCGACACCCTG 360

LGFP TTTAAGGATGATGGAAATTATAAGACAAGGGCAGAAGTGAAGTTTGAAGGTGATACACTG 357

** ***** ** ** ** ** ***** * ** ** ******** ** ** ** ** ***

EGFP GTGAACCGCATCGAGCTGAAGGGCATCGACTTCAAGGAGGACGGCAACATCCTGGGGCAC 420

LGFP GTTAACAGAATTGAGCTGAAAGGCATTGATTTTAAGGAAGATGGAAACATTCTGGGTCAC 417

** *** * ** ******** ***** ** ** ***** ** ** ***** ***** ***

EGFP AAGCTGGAGTACAACTACAACAGCCACAACGTCTATATCATGGCCGACAAGCAGAAGAAC 480

LGFP AAGCTGGAGTACAACTATAATTCTCACAATGTTTACATTATGGCAGATAAGCAGAAGAAT 477

***************** ** ***** ** ** ** ***** ** ***********

EGFP GGCATCAAGGTGAACTTCAAGATCCGCCACAACATCGAGGACGGCAGCGTGCAGCTCGCC 540

LGFP GGAATTAAGGTTAATTTCAAGATTAGACACAACATTGAGGATGGATCTGTCCAACTGGCA 537

** ** ***** ** ******** * ******** ***** ** ** ** ** **

EGFP GACCACTACCAGCAGAACACCCCCATCGGCGACGGCCCCGTGCTGCTGCCCGACAACCAC 600

LGFP GACCATTACCAGCAGAACACCCCTATTGGTGATGGCCCAGTTCTCCTCCCAGATAATCAC 597

***** ***************** ** ** ** ***** ** ** ** ** ** ** ***

EGFP TACCTGAGCACCCAGTCCGCCCTGAGCAAAGACCCCAACGAGAAGCGCGATCACATGGTC 660

LGFP TATCTCCGCACTCAATCTGCTCTGTCCAAAGACCCTAATGAGAAAAGAGACCACATGGTC 657

** ** **** ** ** ** *** ********* ** ***** * ** *********

EGFP CTGCTGGAGTTCGTGACCGCCGCCGGGATCACTCTCGGCATGGACGAGCTGTACAAGTAA 720

LGFP CTCCTGGAGTTTGTGACAGCAGCAGGAATTACTCTGGGAATGGATGAGCTGTACAAGTAA 717

** ******** ***** ** ** ** ** ***** ** ***** ***************

**Protein sequence alignment**

EGFP MVSKGEELFTGVVPILVELDGDVNGHKFSVSGEGEGDATYGKLTLKFICTTGKLPVPWPT 60

LGFP -MSKGEELFTGVVPILVELDGDVNGHKFSVSGEGEGDATYGKLTLKFICTTGKLPVPWPT 59

:**********************************************************

EGFP LVTTLTYGVQCFSRYPDHMKQHDFFKSAMPEGYVQERTIFFKDDGNYKTRAEVKFEGDTL 120

LGFP LVTTLTYGVQCFSRYPDHMKQHDFFKSAMPEGYVQERTIFFKDDGNYKTRAEVKFEGDTL 119

************************************************************

EGFP VNRIELKGIDFKEDGNILGHKLEYNYNSHNVYIMADKQKNGIKVNFKIRHNIEDGSVQLA 180

LGFP VNRIELKGIDFKEDGNILGHKLEYNYNSHNVYIMADKQKNGIKVNFKIRHNIEDGSVQLA 179

************************************************************

EGFP DHYQQNTPIGDGPVLLPDNHYLSTQSALSKDPNEKRDHMVLLEFVTAAGITLGMDELYK* 239

LGFP DHYQQNTPIGDGPVLLPDNHYLRTQSALSKDPNEKRDHMVLLEFVTAAGITLGMDELYK* 238

********************** *************************************

**Supplementary Data S2. Hyaluronidase sequence alignments**

**Gene sequence alignment**

BHU ATGAGCAGACCCCTGGTGATCACCGAGGGCATGATGATCGGCGTGCTGCTGATGCTGGCC 60

BWT ATGTCTCGGCCTCTCGTGATCACGGAAGGGATGATGATTGGAGTGTTGCTAATGCTAGCC 60

*** * ** ** ******** ** ** ******** ** *** **** ***** ***

BHU CCTATCAACGCCCTGCTGCTGGGCTTTGTGCAGAGCACTCCCGACAACAACAAGACCGTG 120

BWT CCGATAAACGCGTTATTACTCGGCTTCGTACAGAGCACCCCCGACAACAACAAAACCGTA 120

** ** ***** * * ** ***** ** ******** ************** *****

BHU CGCGAGTTTAACGTGTACTGGAACGTGCCCACCTTCATGTGCCACAAGTACGGCCTGCGC 180

BWT CGGGAGTTCAACGTTTACTGGAACGTGCCCACCTTTATGTGCCATAAATACGGGCTACGG 180

** ***** ***** ******************** ******** ** ***** ** **

BHU TTTGAGGAGGTGAGCGAGAAGTACGGCATCCTGCAGAACTGGATGGACAAGTTTCGCGGA 240

BWT TTCGAAGAAGTATCGGAGAAATATGGTATTCTACAGAACTGGATGGATAAGTTTCGGGGC 240

** ** ** ** ***** ** ** ** ** ************** ******** **

BHU GAGGAGATTGCCATCCTGTACGACCCTGGCATGTTTCCCGCTCTGCTGAAGGATCCCAAC 300

BWT GAGGAGATCGCGATCCTTTACGACCCTGGAATGTTCCCGGCGTTGCTGAAAGACCCGAAT 300

******** ** ***** *********** ***** ** ** ******* ** ** **

BHU GGCAACGTGGTGGCTCGCAACGGAGGCGTGCCTCAGCTGGGCAACCTGACCAAGCACCTG 360

BWT GGGAACGTGGTGGCGAGGAACGGCGGTGTCCCGCAACTGGGCAATCTCACCAAGCATCTG 360

** *********** * ***** ** ** ** ** ******** ** ******** ***

BHU CAGGTGTTTCGCGACCACCTGATCAACCAGATTCCCGACAAGAGCTTTCCCGGAGTGGGC 420

BWT CAAGTATTTCGGGACCACTTGATCAATCAGATCCCGGACAAGTCGTTTCCCGGCGTGGGG 420

** ** ***** ****** ******* ***** ** ****** ******** *****

BHU GTGATCGACTTTGAGAGCTGGAGACCCATCTTTCGCCAGAACTGGGCTAGCCTGCAGCCC 480

BWT GTGATCGATTTCGAAAGTTGGAGGCCGATATTCAGACAGAACTGGGCCTCCCTCCAGCCT 480

******** ** ** ** ***** ** ** ** * *********** *** *****

BHU TACAAGAAGCTGAGCGTGGAGGTGGTGCGCAGAGAGCACCCTTTCTGGGACGACCAGCGC 540

BWT TACAAGAAACTGTCCGTAGAGGTGGTTCGCCGTGAGCATCCGTTCTGGGACGATCAGAGG 540

******** *** *** ******** *** * ***** ** *********** *** *

BHU GTGGAGCAGGAGGCCAAGAGACGCTTTGAGAAGTACGGCCAGCTGTTCATGGAGGAGACC 600

BWT GTGGAGCAGGAGGCGAAACGAAGGTTCGAGAAATACGGGCAGCTTTTCATGGAGGAGACG 600

************** ** ** * ** ***** ***** ***** **************

BHU CTGAAGGCAGCCAAGCGCATGAGACCTGCTGCCAACTGGGGCTACTACGCCTACCCTTAC 660

BWT TTGAAAGCGGCGAAACGGATGAGGCCGGCCGCCAATTGGGGATACTACGCCTACCCTTAT 660

**** ** ** ** ** ***** ** ** ***** ***** *****************

BHU TGCTACAACCTGACTCCCAACCAGCCCAGCGCCCAGTGCGAGGCCACTACCATGCAGGAG 720

BWT TGCTACAATCTGACGCCGAATCAGCCGAGCGCCCAATGCGAAGCGACCACCATGCAGGAG 720

******** ***** ** ** ***** ******** ***** ** ** ************

BHU AACGACAAGATGAGCTGGCTGTTTGAGAGCGAGGACGTGCTGCTGCCCAGCGTGTACCTG 780

BWT AACGATAAAATGTCGTGGCTGTTCGAGTCGGAAGACGTCCTCCTTCCGTCCGTTTACTTG 780

***** ** *** ******** *** ** ***** ** ** ** *** *** **

BHU CGCTGGAACCTGACCAGCGGCGAGCGCGTTGGACTGGTTGGAGGACGCGTGAAGGAGGCC 840

BWT AGATGGAATCTGACGAGCGGCGAAAGAGTGGGCCTGGTCGGTGGCCGCGTGAAGGAGGCG 840

* ***** ***** ******** * ** ** ***** ** ** **************

BHU CTGAGAATTGCCAGACAGATGACCACTAGCCGCAAGAAGGTGCTGCCCTACTACTGGTAC 900

BWT TTGAGAATAGCGAGGCAAATGACGACCAGCAGGAAGAAGGTTCTACCATATTACTGGTAC 900

******* ** ** ** ***** ** *** * ******** ** ** ** *********

BHU AAGTACCAGGACAGACGCGACACCGACCTGAGCAGAGCCGACCTGGAAGCCACTCTGCGC 960

BWT AAATATCAGGATCGAAGGGACACGGATTTGAGCAGGGCTGACCTCGAGGCAACTTTACGA 960

** ** ***** ** * ***** ** ******* ** ***** ** ** *** * **

BHU AAGATCACCGACCTGGGAGCTGACGGCTTCATCATCTGGGGCAGCAGCGACGACATCAAC 1020

BWT AAAATCACGGACCTCGGCGCCGACGGGTTCATCATTTGGGGAAGTTCCGACGATATAAAC 1020

** ***** ***** ** ** ***** ******** ***** ** ****** ** ***

BHU ACCAAGGCCAAGTGCCTGCAGTTTCGCGAGTACCTGAACAACGAGCTTGGACCTGCCGTG 1080

BWT ACGAAGGCGAAGTGCCTACAATTCAGGGAATACCTGAACAACGAGTTGGGCCCTGCCGTT 1080

** ***** ******** ** ** * ** *************** * ** ********

BHU AAGCGCATTGCCCTGAACAACAACGCCAACGACAGACTGACCGTGGACGTGAGCGTGGAC 1140

BWT AAACGAATCGCGTTGAACAACAACGCGAACGATCGACTGACGGTGGACGTGAGCGTGGAC 1140

** ** ** ** ************* ***** ******* ******************

BHU CAGGTGTAA 1149

BWT CAAGTGTGA 1149

** **** *

**Protein sequence alignment**

BHU MSRPLVITEGMMIGVLLMLAPINALLLGFVQSTPDNNKTVREFNVYWNVPTFMCHKYGLR 60

BWT MSRPLVITEGMMIGVLLMLAPINALLLGFVQSTPDNNKTVREFNVYWNVPTFMCHKYGLR 60

************************************************************

BHU FEEVSEKYGILQNWMDKFRGEEIAILYDPGMFPALLKDPNGNVVARNGGVPQLGNLTKHL 120

BWT FEEVSEKYGILQNWMDKFRGEEIAILYDPGMFPALLKDPNGNVVARNGGVPQLGNLTKHL 120

************************************************************

BHU QVFRDHLINQIPDKSFPGVGVIDFESWRPIFRQNWASLQPYKKLSVEVVRREHPFWDDQR 180

BWT QVFRDHLINQIPDKSFPGVGVIDFESWRPIFRQNWASLQPYKKLSVEVVRREHPFWDDQR 180

************************************************************

BHU VEQEAKRRFEKYGQLFMEETLKAAKRMRPAANWGYYAYPYCYNLTPNQPSAQCEATTMQE 240

BWT VEQEAKRRFEKYGQLFMEETLKAAKRMRPAANWGYYAYPYCYNLTPNQPSAQCEATTMQE 240

************************************************************

BHU NDKMSWLFESEDVLLPSVYLRWNLTSGERVGLVGGRVKEALRIARQMTTSRKKVLPYYWY 300

BWT NDKMSWLFESEDVLLPSVYLRWNLTSGERVGLVGGRVKEALRIARQMTTSRKKVLPYYWY 300

************************************************************

**Supplementary Data S3. FBiTE sequence alignments**

**DNA sequence alignment**

FBiTE ATGGGATGGTCCTGTATTATTCTGTTCCTGGTCGCAACTGCAACTGGCGTCCACTCCCAG 60

FBiTEHU ATGGGCTGGTCCTGCATCATCCTGTTCCTGGTGGCCACCGCCACAGGCGTGCACAGCCAG 60

***** ******** ** ** *********** ** ** ** ** ***** *** ****

FBiTE ATCGTGCTGACCCAGAGCCCAGCCATTATGAGTGCTTCACCCGGCGAAAAGGTGACCATG 120

FBiTEHU ATCGTGCTGACCCAGTCCCCCGCCATCATGTCCGCCAGCCCCGGAGAGAAGGTGACCATG 120

*************** *** ***** *** ** ***** ** ************

FBiTE ACATGTTCCGCCTCTAGTGGGGTCAACTTTATGCACTGGTATCAGCAGAAGAGCGGAACC 180

FBiTEHU ACCTGCAGCGCCAGCTCCGGCGTGAACTTCATGCACTGGTACCAGCAGAAGTCCGGCACC 180

** ** **** ** ** ***** *********** ********* *** ***

FBiTE TCCCCAAAAAGATGGATCTTCGACACAAGCAAACTGGCATCCGGAGTGCCAGCTAGGTTT 240

FBiTEHU AGCCCCAAGAGGTGGATCTTCGACACCAGCAAGCTGGCCTCCGGCGTGCCTGCCAGGTTC 240

*** ** ** ************** ***** ***** ***** ***** ** *****

FBiTE AGCGGATCCGGCTCTGGGACTAGTTACTCACTGACCATTTCAAGCATGGAGGCCGAAGAT 300

FBiTEHU TCCGGCAGCGGCAGCGGCACATCCTACTCCCTGACCATCAGCAGCATGGAAGCCGAGGAT 300

*** **** ** ** ***** ******** ******** ***** ***

FBiTE GCCGCTACCTACTATTGCCAGCAGTGGTCTTTCAATCCCCCTACATTTGGCGGGGGAACT 360

FBiTEHU GCCGCCACCTACTACTGCCAGCAGTGGTCCTTCAATCCTCCCACCTTCGGCGGCGGCACA 360

***** ******** ************** ******** ** ** ** ***** ** **

FBiTE AAGCTGGAGATCAAACGGGGAGGAGGAGGAAGCGGAGGAGGAGGATCCGGAGGCGGGGGA 420

FBiTEHU AAGCTGGAGATCAAGAGGGGCGGCGGCGGCAGCGGAGGAGGAGGAAGCGGAGGAGGCGGC 420

************** **** ** ** ** *************** ****** ** **

FBiTE TCTCAGGTCCAGCTGCAGCAGTCAGGAGCCGAGCTGGCACGACCAGGCGCCAGCGTGAAC 480

FBiTEHU AGCCAGGTGCAGCTGCAGCAGTCCGGCGCCGAGCTGGCCAGGCCTGGAGCCTCCGTGAAC 480

***** ************** ** *********** * ** ** *** *******

FBiTE CTGTCCTGCAAGGCCTCTGGCTACACATTCACTAACAATGGGATCAATTGGCTGAAACAG 540

FBiTEHU CTGAGCTGCAAGGCCAGCGGCTACACCTTCACCAACAATGGCATCAACTGGCTGAAGCAG 540

*** ********** ******** ***** ******** ***** ******** ***

FBiTE CGAACAGGACAGGGCCTGGAGTGGATCGGAGAAATCTACCCCCGGAGCACCAACACACTG 600

FBiTEHU AGGACAGGCCAGGGCCTGGAGTGGATCGGCGAGATCTACCCCAGGTCCACCAATACCCTG 600

* ***** ******************** ** ********* ** ****** ** ***

FBiTE TATAATGAGAAGTTCAAAGGCAAGGCTACTCTGACCGCAGACAGGAGCTCCAACACAGCA 660

FBiTEHU TACAACGAGAAGTTCAAGGGCAAGGCCACCCTGACCGCCGATAGGAGCAGCAACACAGCC 660

** ** *********** ******** ** ******** ** ****** *********

FBiTE TATATGGAGCTGCGCTCTCTGACTAGTGAAGATAGCGCCGTGTACTTTTGCGCACGCACA 720

FBiTEHU TACATGGAGCTGAGGAGCCTGACAAGCGAGGACAGCGCCGTGTACTTCTGTGCCAGGACC 720

** ********* * ***** ** ** ** ************** ** ** * **

FBiTE CTGACTGCTCCCTTCGCATTTTGGGGGCAGGGAACCCTGGTGACAGTCTCCGCTGGAGGA 780

FBiTEHU CTGACCGCCCCTTTCGCCTTCTGGGGCCAGGGCACACTGGTGACAGTGAGCGCCGGCGGC 780

***** ** ** ***** ** ***** ***** ** *********** *** ** **

FBiTE GGAGGAAGCGATATCAAGCTGCAGCAGAGCGGAGCTGAACTGGCACGACCAGGAGCCAGC 840

FBiTEHU GGCGGATCTGACATCAAGCTGCAGCAGAGCGGCGCCGAGCTCGCCAGGCCAGGAGCCTCC 840

** *** ** ******************** ** ** ** ** * ********* *

FBiTE GTGAAAATGTCCTGTAAGACATCTGGCTACACTTTCACCCGATATACTATGCACTGGGTC 900

FBiTEHU GTCAAGATGAGCTGCAAGACCTCCGGCTACACCTTCACCAGGTACACCATGCACTGGGTG 900

** ** *** *** ***** ** ******** ****** * ** ** ***********

FBiTE AAGCAGCGACCTGGACAGGGACTGGAGTGGATCGGATACATTAATCCAAGCCGGGGCTAC 960

FBiTEHU AAGCAGAGGCCCGGCCAGGGCCTCGAGTGGATCGGCTACATCAACCCCAGCAGAGGCTAC 960

****** * ** ** ***** ** *********** ***** ** ** *** * ******

FBiTE ACAAACTACAACCAGAAGTTTAAAGACAAGGCTACCCTGACTACCGATAAGAGCTCCTCT 1020

FBiTEHU ACCAACTACAACCAGAAGTTCAAGGACAAGGCCACCCTGACCACCGACAAGTCCAGCAGC 1020

** ***************** ** ******** ******** ***** *** * *

FBiTE ACAGCATACATGCAGCTGAGTTCACTGACTTCTGAGGACAGTGCAGTGTACTATTGCGCC 1080

FBiTEHU ACAGCCTACATGCAGCTGAGCAGCCTGACCAGCGAGGACTCCGCCGTGTACTACTGCGCC 1080

***** ************** ***** ****** ** ******** ******

FBiTE AGATACTATGACGATCATTACTGTCTGGATTATTGGGGACAGGGCACAACTCTGACAGTG 1140

FBiTEHU AGGTACTACGATGATCACTACTGTCTGGATTACTGGGGCCAGGGAACAACCCTGACAGTG 1140

** ***** ** ***** ************** ***** ***** ***** *********

FBiTE AGCTCCGTCGAAGGAGGATCAGGAGGAAGCGGAGGATCCGGAGGGTCTGGAGGCGTGGAC 1200

FBiTEHU TCCAGCGTGGAGGGCGGCAGCGGCGGATCTGGAGGAAGCGGTGGCAGCGGCGGCGTGGAT 1200

* *** ** ** ** ** *** ****** *** ** ** ********

FBiTE GATATCCAGCTGACTCAGTCCCCAGCCATTATGTCCGCTTCTCCCGGCGAGAAAGTGACA 1260

FBiTEHU GATATCCAGCTGACCCAGAGCCCTGCCATCATGAGCGCCTCCCCCGGCGAGAAGGTGACA 1260

************** *** *** ***** *** *** ** *********** ******

FBiTE ATGACTTGCCGGGCCTCTAGTTCAGTCTCTTACATGAATTGGTATCAGCAGAAAAGTGGC 1320

FBiTEHU ATGACCTGTAGAGCCAGCTCCTCCGTGAGCTACATGAATTGGTATCAACAAAAGTCCGGC 1320

***** ** * *** ** ** ***************** ** ** ***

FBiTE ACCTCACCAAAGAGATGGATCTACGACACCAGTAAGGTGGCCAGCGGGGTCCCCTATAGG 1380

FBiTEHU ACCAGCCCCAAGAGGTGGATCTATGATACCTCCAAGGTGGCCTCCGGCGTCCCTTACAGG 1380

*** ** ***** ******** ** *** ********* *** ***** ** ***

FBiTE TTTAGTGGCTCCGGAAGCGGCACCTCCTACTCTCTGACAATTAGCTCCATGGAGGCAGAA 1440

FBiTEHU TTCAGCGGCAGCGGAAGCGGCACAAGCTACTCCCTGACCATCAGCAGCATGGAGGCCGAG 1440

** ** *** ************ ****** ***** ** *** ********* **

FBiTE GATGCCGCTACCTACTATTGTCAGCAGTGGTCTAGTAACCCTCTGACATTCGGGGCTGGG 1500

FBiTEHU GATGCTGCCACATACTACTGCCAGCAGTGGAGCTCCAACCCTCTGACATTCGGCGCCGGC 1500

***** ** ** ***** ** ********* ***************** ** **

FBiTE ACCAAACTGGAGCTGAAGGATTACAAGGACGACGACGACAAGTAATAAA 1549

FBiTEHU ACAAAGCTCGAACTGAAGGACTACAAGGACGACGATGACAAGTAATAAA 1549

** ** ** ** ******** ************** *************

**Protein sequence alignment**

FBiTE MGWSCIILFLVATATGVHSQIVLTQSPAIMSASPGEKVTMTCSASSGVNFMHWYQQKSGT 60

FBiTEHU MGWSCIILFLVATATGVHSQIVLTQSPAIMSASPGEKVTMTCSASSGVNFMHWYQQKSGT 60

************************************************************

FBiTE SPKRWIFDTSKLASGVPARFSGSGSGTSYSLTISSMEAEDAATYYCQQWSFNPPTFGGGT 120

FBiTEHU SPKRWIFDTSKLASGVPARFSGSGSGTSYSLTISSMEAEDAATYYCQQWSFNPPTFGGGT 120

************************************************************

FBiTE KLEIKRGGGGSGGGGSGGGGSQVQLQQSGAELARPGASVNLSCKASGYTFTNNGINWLKQ 180

FBiTEHU KLEIKRGGGGSGGGGSGGGGSQVQLQQSGAELARPGASVNLSCKASGYTFTNNGINWLKQ 180

************************************************************

FBiTE RTGQGLEWIGEIYPRSTNTLYNEKFKGKATLTADRSSNTAYMELRSLTSEDSAVYFCART 240

FBiTEHU RTGQGLEWIGEIYPRSTNTLYNEKFKGKATLTADRSSNTAYMELRSLTSEDSAVYFCART 240

************************************************************

FBiTE LTAPFAFWGQGTLVTVSAGGGGSDIKLQQSGAELARPGASVKMSCKTSGYTFTRYTMHWV 300

FBiTEHU LTAPFAFWGQGTLVTVSAGGGGSDIKLQQSGAELARPGASVKMSCKTSGYTFTRYTMHWV 300

************************************************************

FBiTE KQRPGQGLEWIGYINPSRGYTNYNQKFKDKATLTTDKSSSTAYMQLSSLTSEDSAVYYCA 360

FBiTEHU KQRPGQGLEWIGYINPSRGYTNYNQKFKDKATLTTDKSSSTAYMQLSSLTSEDSAVYYCA 360

************************************************************

FBiTE RYYDDHYCLDYWGQGTTLTVSSVEGGSGGSGGSGGSGGVDDIQLTQSPAIMSASPGEKVT 420

FBiTEHU RYYDDHYCLDYWGQGTTLTVSSVEGGSGGSGGSGGSGGVDDIQLTQSPAIMSASPGEKVT 420

************************************************************

FBiTE MTCRASSSVSYMNWYQQKSGTSPKRWIYDTSKVASGVPYRFSGSGSGTSYSLTISSMEAE 480

FBiTEHU MTCRASSSVSYMNWYQQKSGTSPKRWIYDTSKVASGVPYRFSGSGSGTSYSLTISSMEAE 480

************************************************************

FBiTE DAATYYCQQWSSNPLTFGAGTKLELKDYKDDDDK 514

FBiTEHU DAATYYCQQWSSNPLTFGAGTKLELKDYKDDDDK 514

**********************************
